# Attitudes, experiences, and preferences of ophthalmic professionals regarding routine use of patient-reported outcome measures in clinical practice

**DOI:** 10.1371/journal.pone.0243563

**Published:** 2020-12-04

**Authors:** Alexandra O. Robertson, Valerija Tadić, Jugnoo S. Rahi

**Affiliations:** 1 Population, Policy & Practice Research and Teaching Department, UCL Great Ormond Street (GOS) Institute of Child Health, London, United Kingdom; 2 School of Human Sciences, University of Greenwich, London, United Kingdom; 3 Great Ormond Street Hospital NHS Foundation Trust, London, United Kingdom; 4 National Institute for Health Research (NIHR) Biomedical Research Centre at Moorfields Eye Hospital NHS Foundation Trust and UCL Institute of Ophthalmology, London, United Kingdom; 5 Ulverscroft Vision Research Group, London, United Kingdom; Queensland University of Technology, AUSTRALIA

## Abstract

**Background/Objectives:**

Routine use of patient-reported outcome measures (PROMs) to assess quality of health care systems is mandated in many countries and has been implemented successfully in many specialities. Ophthalmology currently lags behind. To support and inform future implementation, we investigated paediatric ophthalmic clinicians’ experience of, and future training needs for, using child-appropriate vision PROMs and their views about the barriers and enablers to future routine implementation in clinical practice.

**Methods:**

We conducted a pilot study, using an online survey to elicit the experience, attitudes, training needs and perceptions of barriers and enablers to routine PROMs use of ophthalmic health professionals in the Paediatric Ophthalmology Department at Great Ormond Street Hospital, London. A focus-group was undertaken to discuss survey results and preferences regarding presentation of PROM data. Analysis comprised descriptive statistics, presented alongside complementary qualitative data.

**Results:**

Eighteen clinicians in the department completed the survey. Twenty-seven took part in the focus group. Clinicians had limited experience of using PROMs but high confidence in the potential positive impact on communication with patients, monitoring chronic conditions and clinical decision-making. Clinicians identified operational issues (collection and analysis of data) and impact (interpretation and application of data) as the two key areas for consideration. Training and information requirements before implementation were clearly articulated, alongside the benefits of using digital/electronic data capture ahead of consultations to allow efficiency and automated analysis, and presentation in an appropriate visual format alongside clinical data to ensure meaningful use.

**Conclusion:**

The findings of this pilot study of ophthalmic clinicians working in a specialist paediatric ophthalmology department, suggest that ophthalmic clinicians recognise the potential benefits of routine PROMs use in clinical practice. Together with existing literature outside ophthalmology relating to overcoming barriers and exploiting enablers to routine implementation, findings may be applicable in planning routine PROM implementation in paediatric ophthalmology.

## Introduction

High quality healthcare has three domains: safety, effectiveness and positive patient experience [1, 2]. Patient-reported outcome measures (PROMs) are now established as key tools for measuring effectiveness. Routine use of PROMs is widely advocated [3–5] and has been used to assess and improve the quality of healthcare in many countries [6]. In the UK National Health Service (NHS), routine PROM use was initially mandated for four high volume ‘beacon’ surgical procedures in adults with the expectation that improved subjective well-being would reflect high quality care [7]. As yet, routine use of PROMs to measure the quality of ophthalmic health services is not mandated. PROMs are recognised as particularly valuable adjuncts to clinical assessment in chronic conditions, where clinical parameters may change little but the impact on the lives of affected individuals may vary significantly. Thus in the context of chronic conditions, promoting health-related quality of life becomes an important focus of healthcare [8].

Childhood visual impairment is a prime exemplar of a chronic health state, with a profound and dynamic impact on subjective well-being and activities of daily living. The impact on development and participation during childhood are well described, including the risk of delayed social, cognitive and emotional milestones [9, 10], and limitations to age-appropriate activities [11]. As children grow up, the impact of visual impairment may change, due to a combination of disease progression, change in clinical treatment/intervention and/or the child’s adaptation to the functional limitations imposed by visual impairment. Thus PROMs have significant potential value in clinical practice, affording rich insight into the *broader* impact of visual impairment upon aspects of daily life that are not captured by clinical assessment.

Until recently one key barrier to routine PROMs use in ophthalmology has been a dearth of robust vision PROMs [12]. With a burgeoning vision PROMs industry, it is time to address another gap in the evidence base, the lack of understanding of the barriers and enablers to routine use of vision PROMs from the perspectives of ophthalmic clinicians.

We report a novel investigation, using our two child/young person vision PROMs [13–16] as ‘model’ instruments, of ophthalmic clinicians’ prior experience of, and future training needs for, using PROMs and their views about the barriers and enablers to future implementation in paediatric ophthalmology practice.

## Materials and methods

This pilot service development and quality improvement study was approved by the National Health Service Research Ethics Committee for University College London Great Ormond Street Institute of Child Health and Great Ormond Street Hospital, London, UK (REC reference: 17/LO/1484). The study followed the tenets of the Declaration of Helsinki.

### Sample

A voluntary sample of clinicians based in the Department of Ophthalmology at Great Ormond Street Hospital, London UK, an internationally leading children’s hospital. All members of the patient-facing multi-professional clinical team, comprising ophthalmologists, orthoptists, optometrists, clinical vision scientists, nurses, and an eye clinic liaison officer, were invited by the leading researcher (AR) to participate in this study. Due to the nature of the study design (i.e. a study of all ‘patient-facing’ staff in a single department), no exclusion criteria were used.

### Procedure

Participants were recruited through verbal invitation during a clinical teaching session at the hospital, which took place in July 2018, attended by 31 clinicians in the Department. The aims of the project were presented alongside the NHS policy framework and context for routine use of PROMs. In order to understand experience and perspectives relevant to paediatric ophthalmology specifically, we used our previously developed instruments capturing the distinct but complementary outcomes of vision-related quality of life (the VQoL_CYP) [13, 14, 16] and functional vision (the FVQ_CYP) [15, 17], as two exemplar child-vision PROMs (available for download at https://xip.uclb.com/ct/healthcare_tools/). An overview of the VQoL_CYP and FVQ_CYP was presented by way of an update, as they were already familiar to most participants since the research programme that developed these PROMs was based at the hospital’s partner institution, the UCL Great Ormond Street Institute of Child Health. Several members of the broader research team in which the VQoL_CYP and FVQ_CYP were developed were also present at the clinical teaching session. Following this briefing session, an online survey was distributed by email to the whole clinical team.

The survey was constructed using RedCap software [18]. A combination of closed- and open-ended questions were used that took account of the existing literature outside ophthalmology relating to routine PROM use. The survey elicited prior experience of using PROMs, self-assessed further training/information needs, level of confidence in discussing PROMs with patients, and agreement with well-known benefits and barriers of using PROMs routinely in clinical practice (closed-ended questions are presented in Tables 1 and 2, see S1 Appendix for the full survey). A brief description of the VQoL_CYP [13, 14, 16] and FVQ_CYP [15, 17] instruments preceded the survey. The survey was piloted with a clinical member of the research team who was not part of the clinical department, with an aim to identify any improvements to the wording or presentation of individual questions. Participants submitted their responses anonymously, to encourage candid responses. Given the relatively small size of the clinical department (which is very well known in the UK) and to avoid any risk of disclosure, we deliberately did not collect potentially identifiable information such as participants’ gender, age, experience in clinical practice or their specific role in the department. Three reminders to participate were sent to the whole department over the course of 3 months.

**Table 1:** Clinicians’ self-reported experience, training needs, and preferences for viewing PROM data.

Question	Response (n (%))
**Do you have any professional experience using PROMs**	
*Yes*	4 (22.2)
*No*	14 (77.8)
**Which of the following areas would you like to receive further training/information? (Select all that apply)**	
*What PROMs are*	7 (38.9)
*The benefits of using PROMs*	9 (50)
*The challenges of using PROMs*	10 (55.6)
*How to choose which PROM to use*	15 (83.3)
*How to administer PROMs*	10 (55.6)
*How to calculate PROM scores*	13 (72.2)
*How to interpret PROM scores*	16 (88.9)
**How would you prefer to view PROM scores? (Select one answer)**	
*Numeric presentation*, *e*.*g*. *score = 49*	5 (27.8)
*Visual presentation*, *e*.*g*. *score shown in graph*	9 (50)
*Both numeric and visual*	4 (22.2)

**Table 2:** Agreement and disagreement with benefits and barriers to using PROMs in routine clinical practice.

Question	Response options (n (%))				
**How strongly do you agree or disagree with the following statements about the benefits of using PROMs in Paediatric Ophthalmology services?**	**Strongly agree**	**Agree**	**Neither agree nor disagree**	**Disagree**	**Strongly disagree**
*Using PROMs would help me make clinical decisions about my patients*	4 (22.2)	9 (50)	5 (27.8)	0	0
*Using PROMs would help me detect problems and concerns that clinical assessments would not identify*	6 (33.3)	9 (50)	2 (11.1)	1 (5.6)	0
*Using PROMs would help me monitor my patient’s condition*	4 (22.2)	9 (50)	3 (16.7)	2 (11.1)	0
*Using PROMs would help me monitor my patient’s response to treatment(s)*	3 (16.7)	10 (55.6)	5 (37.8)	0	0
*Using PROMs would help me communicate better with my patients and their parents/caregivers*	6 (33.3)	10 (55.6)	2 (11.1)	0	0
*Using PROMs would help me monitor the quality of care I am providing to all my patients*	4 (22.2)	11 61.1)	3 (16.7)	0	0
*Using PROMs would help my patients and their parents/caregivers make decisions about their health*	7 (38.9)	9 (50)	2 (11.1)	0	0
*Using PROMs would improve patient satisfaction*	6 (33.3)	9 (50)	3 (16.7)	0	0
**How strongly do you agree or disagree with the following statements about the barriers to/disadvantages of using PROMs?**	**Strongly agree**	**Agree**	**Neither agree nor disagree**	**Disagree**	**Strongly disagree**
*I do not have enough time in my clinics to use PROMs*	2 (11.1)	8 (44.4)	6 (33.3)	2 (11.1)	0
*PROM outcomes are not relevant to my clinical decisions*	0	1 (5.6)	6 (33.3)	8 (44.4)	3 (16.7)
*PROMs do not provide any additional information*	0	1 (5.6)	1 (5.6)	11 (61.1)	5 (27.8)
*Using PROMs may have a negative impact on my relationship with my patients*	1 (5.6)	0	0	11 (61.1)	5 (27.8)
*Using PROMs would encourage patients to discuss aspects of health which are beyond my control*	1 (5.6)	4 (22.2)	5 (27.8)	7 (38.9)	1 (5.6)
*Patients might get distressed when they complete PROMs*	0	4 (22.2)	8 (44.4)	5 (27.8)	1 (5.6)

In a follow-up/feedback session, the results of the survey were presented to the clinical team. A one hour long focus group discussion was led by researchers (AR and JR), who are experienced in collecting qualitative data, to enable a ‘deep dive’ into the findings including development of a consensus on the optimal approach to presenting analysed PROMs data to clinicians. Qualitative data were audio recorded.

### Analysis

Quantitative analysis comprised descriptive statistics using SPSS [19].

Qualitative data from open-ended survey items and the focus group discussion were transcribed and entered into NVivo [20]. Data were analysed by two researchers (AR and JR) using qualitative thematic analysis, including open and axial coding techniques [21], to identify key themes, derived from the data, which were then cross-referenced with quantitative findings.

## Results

Eighteen clinicians (47% of the clinical department) completed the survey. Twenty-seven took part in the focus group discussion, representing every different ‘patient-facing’ professional group within the department.

Three themes were derived inductively, based on qualitative analysis: *interpretation of PROM data*, *responsibilities for action*, and *optimal PROM presentation*. These were cross-referenced with quantitative findings, and are presented as complementary data.

As shown in Table 1, only a minority (22.2%) of participants had any experience of using PROMs. Various training and information needs were identified, with a need for training in how to choose the best PROM and how to interpret PROM scores being the most common (>80% respondents). Half or more also identified their need for better understanding of both the benefits and challenges of using PROMs.

Most participants preferred to view purely visual representations of PROM data (versus numeric scores) (Table 1), with some pre-coding:

“…a traffic light system with red getting worse.”

Qualitative data analysis revealed a clear preference for simple presentation formats alongside objective assessments of visual function to support discussions with patients and their families. Flexibility in presentation of PROM data, enabling both overall scores and, where sought, individual item scores to be viewed over time (e.g. a scatterplot) with the option to *“dig deeper”* into individual item scores, was deemed optimal for facilitating interpretation.

**Fig 1:**
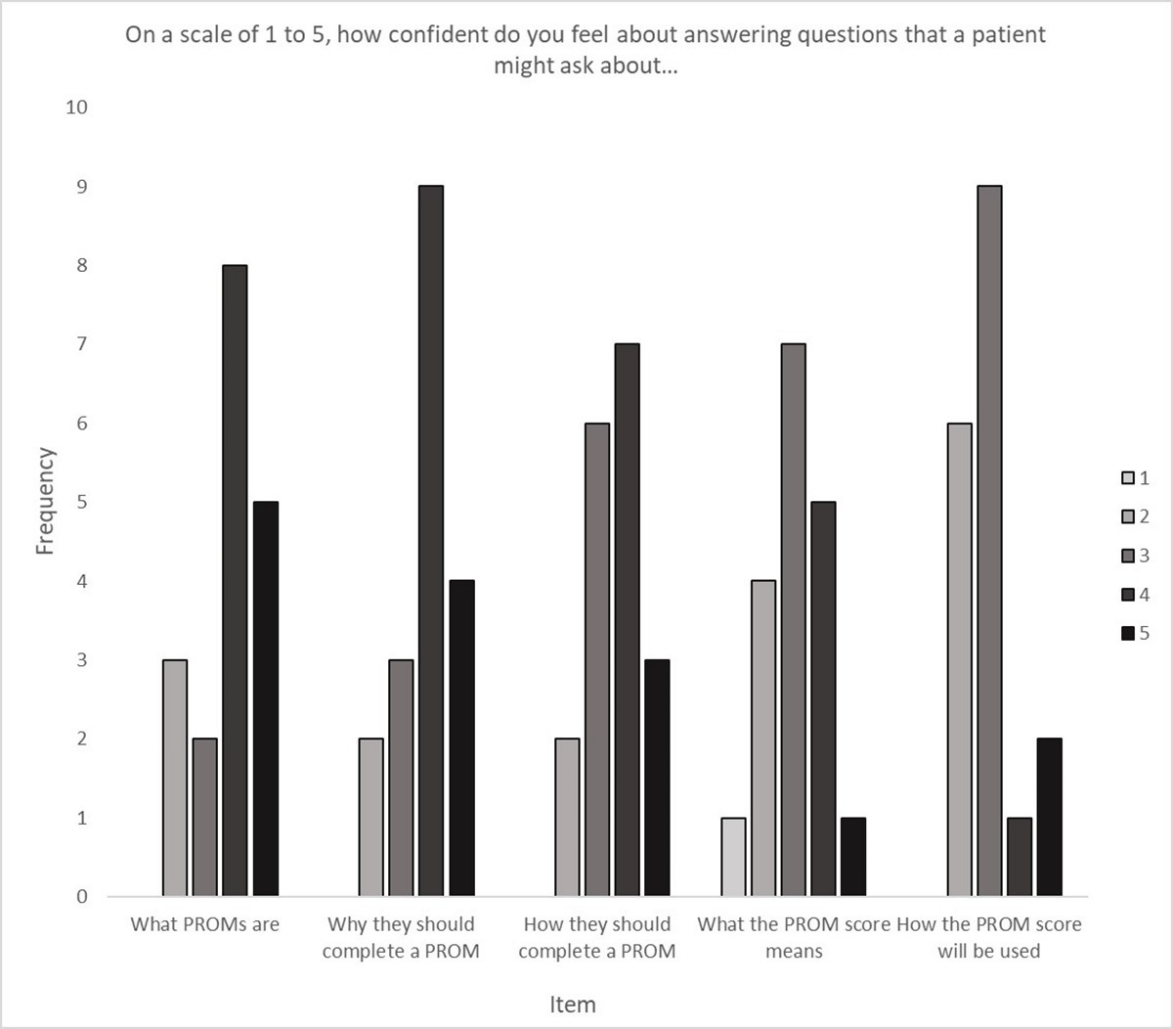
Clinicians’ self-reported confidence to answer hypothetical questions a patient might ask about PROMs.

As shown in Fig 1, clinicians felt most confident about explaining to their patients what PROMs are and why their patients should complete them, but less confident about explaining what scores meant and how they would be used. Complementary qualitative data revealed concerns about interpreting findings in the context of parental influence or missing data. Incorporating PROM items into electronic patient records, with supporting manuals and embedded algorithms to allow immediate analysis were viewed as facilitating accurate interpretation:

“Tablet and web or cloud based collection systems with immediate analytics, such as scoring would be ideal. It would be great if these could be incorporated into electronic patient records.”

As shown in Table 2, the majority of participants agreed with the well-established potential benefits to using PROMs, notably that PROMs would be useful when i) making clinical decisions, ii) detecting problems and concerns that clinical assessments would not identify, iii) monitoring a patient’s condition and response to treatment, and iv) improving communication and joint decision-making with patients, and their families.

Equally, participants endorsed the commonly reported barriers to routine use by clinicians in other specialties, in particular the risk that using PROMs would encourage patients to discuss aspects of health beyond the control of the clinicians. Some clinicians emphasised this perspective within the focus group, discussing, in more detail, the possible disclosure by patients of psychosocial, and emotional issues. Clinicians’ raised some concerns about their responsibility for action.

“*Are we responsible to act on these issues*? *When does it become a child protection issue*?*”*

There was consensus that *“pre-screening”* of PROMs data by one member of the clinical team with expertise in psychosocial and emotional issues associated with visual impairment, such as the Eye Clinic Liaison Officer, before a clinical review, was the optimal approach:

“These are valuable tools that the Eye Clinic Liaison Officer would benefit from having access to and advising me of the results from, especially if they highlight areas of concern.”

## Discussion

From this pilot study of the views and experiences of paediatric ophthalmic professionals regarding routine use of PROMs, it is clear that clinicians value the benefits of embedding routine use of vision PROMs in ophthalmic practice to improve their understanding and ability to monitor the impact of eye disease and its treatment on their patients and to enhance communications and joint decision-making with their patients and their families. The need for further training and information before implementation were clearly articulated alongside the need to find ways of allowing PROM data to be efficiently collected, analysed and reviewed before being presented in an appropriate format alongside clinical data to ensure meaningful use.

We used a mixed methods approach to understand the perspectives of clinicians working in our tertiary paediatric ophthalmology service i.e. serving the UK population of children and young people with visual impairment or blindness who are the intended users of the VQoL_CYP [13, 14, 16] and FVQ_CYP [15, 17]. This pilot study was designed to ascertain preliminary findings which will be useful to taking the next steps in routine implementation of PROMs in paediatric ophthalmology. Thus, whilst the sample size was adequate for this primary purpose of the study, it precluded formal statistical analyses, for example the relationship between clinicians’ experience and attitudes. Equally, the available resources including clinicians’ time, precluded in-depth individual interviews which could have allowed finer granularity of qualitative data.

Participants in this study were clinicians within a hospital that is the partner to the research institution of the study team, and therefore potentially in a particularly good position to reflect, subjectively and accurately, on their experience of PROMs, and future routine use in an environment which they are extremely familiar with. However, only a minority had prior experience of using any vision PROMs. Whilst it is possible that participants’ prior familiarity with the overarching PROMs research programme might have influenced their participation in the study, as well as their responses, for example their positive attitudes towards using PROMs, it is notable that concerns were also identified. These warrant careful consideration, particularly issues surrounding the interpretation of PROM data which, if done incorrectly, could have serious clinical consequences. Throughout the research processes we tried to minimise any possible bias to ensure the study findings were reliable, encouraging participants to be as open as possible, and ensuring anonymity of data (collected in the survey). We also acknowledge that the single site, specialist setting in which this study took place and use of child-appropriate vision PROMs as exemplars, may preclude direct generalisations of the study findings to other ophthalmic clinical settings. Nevertheless, this study provides some important, preliminary information of generic value in ophthalmology, and notably, is anchored by the existing literature outside ophthalmology. Thus we believe the novel findings of this study to be valid and useful in planning future routine PROMs use in ophthalmology in other settings.

Despite limited personal experience of using PROMs, most clinicians participating in this study recognised powerful benefits that are already evidenced in the literature outside ophthalmology, in particular improvements in patient-doctor communication [22] and empowering patients’ to make decisions about their health/treatment [23]. The growing literature points also to other benefits including improved characterisation of diagnoses [24], ability to capture concerns beyond the scope of functional clinical assessments [24] and usefulness in monitoring long-term conditions [25] Our findings suggest that implementation of routine use of PROMs in ophthalmology does not require further research to identify ‘ophthalmology-specific’ benefits but rather that existing experience and literature on potential benefits could be utilised to provide information and plan training for ophthalmic clinicians. For example a reported intervention model in paediatrics (incorporating educational, epidemiological, behavioural, organisational, and social interaction approaches) utilising online PROM administration accompanied by generic training about PROMs for clinicians before observing (via DVD) others using PROMs [26] could be translated into paediatric ophthalmology after due consideration of the population of patients being served [27] and training in how to choose the most appropriate PROM [28].

Whilst the generic literature points to some concerns amongst clinicians about whether routine use of PROMs truly makes a difference to clinical outcomes [29], the finding that the majority of clinicians in our study assigned particular value to PROMs in clinical decision-making is in keeping with other health professionals [6, 30]. This most likely reflects understanding that the *way* in which routine PROM use is undertaken is critical to realising its full benefits. Our participants views regarding barriers and enablers aligned with two key themes in the extant literature: operationalisation (how best to collect and incorporate data) and impact (how best to interpret and act on the data to change patient care) [31]. The empirical literature shows that use of digital technology and electronic systems to administer PROMs and manage the data is efficient and effective [4]. Moreover if patients are able to complete PROMs ahead of their consultation and can also view their own longitudinal PROM data, this may allow priorities for discussion at the upcoming consultation to be identified and allow better pre-planning of key decision-making outpatient appointments [4, 32, 33]. This approach could work well in NHS ophthalmology services, where there is generally already a member of the team (e.g. Eye Clinic Liaison Officer (ECLO) or equivalent) who is ideally placed to review and discuss the PROM data with patients before briefing, as required, the managing clinician. With regards to presenting PROM data, this study clearly identifies a visual format embedded within an electronic patient record system would be optimal, facilitating clinicians’ interpretation and minimising time spent viewing data. Such a system needs to be flexible, offering users the ability to switch between graphical summaries and a deep dive into raw data. The global drive towards electronic patient record systems provides the ideal vehicle for integration of PROMs into routine ophthalmic care, to provide an integrated and flexible platform for PROMs collection as has already been achieved in other paediatric areas [34, 35].

Whilst ophthalmology is not yet on a par with other clinical specialities in terms of either availability of robust PROMs [12] or routine implementation [28, 36, 37], we suggest that there is cause for optimism based on our study findings. The findings of this pilot study show that the potential benefits of routine PROM use are recognised by ophthalmic clinicians and that they have an appetite to learn about how to choose and use the most appropriate PROMs. They also suggest that the existing literature outside ophthalmology relating to overcoming barriers and exploiting enablers to routine implementation may be applicable to planning implementation in ophthalmology.

## Supporting information

S1 Appendix (DOCX)
