# Supplementary material for: Attitudes, experiences, and preferences of ophthalmic professionals regarding routine use of patient-reported outcome measures in clinical practice
Source: PLoS One. 2020 Dec 4;15(12):e0243563. doi: 10.1371/journal.pone.0243563 (PMC7717508; doi:10.1371/journal.pone.0243563)
Supplement: S1 Appendix — (DOCX) [file pone.0243563.s001.docx]

**A survey of the optimal routine use of Patient Reported Outcome Measures (PROMs) in Paediatric Ophthalmology**

Dear Colleague,

We would like to ask you to take part in this study, by completing this survey about patient-reported outcome measures (PROMs).

As you will know, PROMs are questionnaires which can be used to collect information on patient’s perspectives of their illness, its impact upon them and the impact of any treatment.

In the NHS, PROMs are currently mandatory outcomes for hip and knee surgery and have been described as the ‘gold standard’ for reporting on health outcomes. Although they are not yet mandated in paediatric ophthalmology services, they are considered valuable outcomes for understanding the impact of visual impairment.

We have developed two PROMs: the first captures vision-related quality of life and the second measures functional vision. We have developed two versions of each which are suitable for use by children (aged 8-12 years) and by young people (aged 13-17 years) respectively. These PROMs contain 20-38 items and require patients to use a rating scale to quantify their response. These are ‘summed’ to produce a summary score.

We are now undertaking a pilot study to find out the best way to use these PROMs. We are interested in your experience and attitude towards using PROMs routinely within Ophthalmology services at Great Ormond Street Hospital. This information will help planning of how to incorporate PROMs and the training resources required to support clinicians using the PROMs.

**1. How would you prefer to view PROM summary scores? *Select all that apply.***

- Numeric presentation of individual item/summary scores
- Visual presentation, for example, graphs showing scores across items or different time points

**Please tell us any other ways you would like to view PROM summary scores.**

**---------------------------------------------------------------------------------------------------------------------------------------------------------------------------------------------------------------------------------------------------------------------------------------------------------------------------------------------------------------------------------**

**2. Do you have any professional experience using PROMs?**

- Yes
- No

**3. On a scale of 1 to 5, how confident do you feel about answering questions that a patient might ask about…**

|  | 1 (not at all confident) | 2 | 3 | 4 | 5 (Very confident) |
| --- | --- | --- | --- | --- | --- |
| *What PROMs are?* |  |  |  |  |  |
| *Why they should complete a PROM questionnaire?* |  |  |  |  |  |
| *How they should complete a PROM questionnaire?* |  |  |  |  |  |
| *What the PROM score means?* |  |  |  |  |  |
| *How the PROM score will be used?* |  |  |  |  |  |

**4. Which of the following areas would you like to receive further training/information? *Select all that apply.***

| What PROMs are |  |
| --- | --- |
| The benefits of using PROMs |  |
| The challenges of using PROMs |  |
| How to choose which PROM to use |  |
| How to administer PROMs |  |
| How to calculate PROM scores |  |
| How to interpret PROM scores |  |

**Please tell us about any other areas of training you feel would be helpful.**

**---------------------------------------------------------------------------------------------------------------------------------------------------------------------------------------------------------------------------------------------------------------------------------------------------------------------------------------------------------------------------------**

**5. How strongly do you agree or disagree with the following statements about the benefits of using PROMs in Paediatric Ophthalmology services?**

| Using PROMs… | Strongly agree | Agree | Neither agree nor disagree | Disagree | Strongly disagree |
| --- | --- | --- | --- | --- | --- |
| *…would help me make clinical decisions about my patients.* |  |  |  |  |  |
| *…would help me detect problems and concerns that clinical assessments would not identify.* |  |  |  |  |  |
| *…would help me monitor my patients’ condition.* |  |  |  |  |  |
| *…would help me monitor my patients’ response to treatment(s).* |  |  |  |  |  |
| *…would help me communicate better with my patients and their parents/caregivers.* |  |  |  |  |  |
| *…would help me monitor the quality of care I am providing to all my patients.* |  |  |  |  |  |
| *…would help my patients and their parents/caregivers make decisions about their health.* |  |  |  |  |  |
| *…would improve patient satisfaction.* |  |  |  |  |  |

**Please tell us about any other ways you think Paediatric Ophthalmology services may benefit from using PROMs routinely.**

**---------------------------------------------------------------------------------------------------------------------------------------------------------------------------------------------------------------------------------------------------------------------------------------------------------------------------------------------------------------------------------**

**6. How strongly do you agree with the following statements about the barriers to/disadvantages of using PROMs in clinics?**

|  | Strongly agree | Agree | Neither agree nor disagree | Disagree | Strongly disagree |
| --- | --- | --- | --- | --- | --- |
| I do not have enough time in my clinics to use PROMs. |  |  |  |  |  |
| PROM outcomes are not relevant to my clinical decisions. |  |  |  |  |  |
| PROMs do not provide any additional information. |  |  |  |  |  |
| Using PROMs may have a negative impact on my relationship with my patients. |  |  |  |  |  |
| Using PROMs would encourage patients to discuss aspects of health which are beyond my control. |  |  |  |  |  |
| Patients might get distressed when they complete PROMs*.* |  |  |  |  |  |

**Please tell us about any other concerns you may have about using PROMs in your clinics.**

**---------------------------------------------------------------------------------------------------------------------------------------------------------------------------------------------------------------------------------------------------------------------------------------------------------------------------------------------------------------------------------**

**7. Please describe any resources you feel are needed before you can use PROMs in your clinics.**

**---------------------------------------------------------------------------------------------------------------------------------------------------------------------------------------------------------------------------------------------------------------------------------------------------------------------------------------------------------------------------------**

**End of survey**

**Use this space if you would like to add any additional comments, or expand on any of your answers.**

**---------------------------------------------------------------------------------------------------------------------------------------------------------------------------------------------------------------------------------------------------------------------------------------------------------------------------------------------------------------------------------**

**---------------------------------------------------------------------------------------------------------------------------------------------------------------------------------------------------------------------------------------------------------------------------------------------------------------------------------------------------------------------------------**

**---------------------------------------------------------------------------------------------------------------------------------------------------------------------------------------------------------------------------------------------------------------------------------------------------------------------------------------------------------------------------------**

**---------------------------------------------------------------------------------------------------------------------------------------------------------------------------------------------------------------------------------------------------------------------------------------------------------------------------------------------------------------------------------**
